# An Unsupervised Learning Approach for Multimodal Low Back Pain Stratification

**DOI:** 10.1097/BRS.0000000000005593

**Published:** 2025-12-22

**Authors:** Narasimharao Kowlagi, Eveliina Heikkala, Simo Saarakkala, Jaro Karppinen, Aleksei Tiulpin

**Affiliations:** a Research Unit of Health Sciences and Technology, University of Oulu; b Wellbeing Services County of South Karelia, Lappeenranta; c Dept. of Diagnostic Radiology, University Oulu Hospital, Oulu, Finland

**Keywords:** low back pain, patient stratification, unsupervised learning, deep learning, MRI biomarkers, STarTBack, Örebro

## Abstract

**Study Design.:**

Cross-sectional study.

**Objective.:**

This study proposes a novel stratification framework for individuals with low back pain (LBP). The method integrates Northern Finland Birth Cohort data comprising imaging biomarkers from deep learning (DL)-based analysis of lumbar spine MRI with the data on smoking status, demographics (sex and BMI), self-reported data from Örebro Musculoskeletal Pain Screening Questionnaire (ÖMPSQ) short and the STarT Back Tool (SBT). Furthermore, the utility of this stratified approach was validated by demonstrating a superior net benefit compared with “treat-all” strategy.

**Background.:**

Current risk stratification for individuals with LBP relies on ÖMPSQ short and SBT among others. While these tools are invaluable for capturing psychosocial characteristics predictive of future disability and functional outcomes, LBP’s multifactorial nature necessitates a more comprehensive framework for effective risk stratification.

**Materials and Methods.:**

A method for multimodal unsupervised patient stratification has been developed that allows for the integration of imaging biomarkers of disc degeneration (DD) and facet tropism (FT), extracted using DL models, with nonimaging data. The framework utilized robust K-Means clustering to stratify individuals. Clusters were characterized using LBP frequency and bothersomeness, and their robustness was validated with a multi-class logistic regression model. Net benefit was assessed through decision curve analysis.

**Results.:**

Three distinct subgroups were characterized by LBP frequency and bothersomeness. One subgroup was dominated by psychosocial characteristics (psychosocial risk *P *< 0.05), the second by physical degenerative changes (DD *P *< 0.05), and the third by a mix of both. Predictive models for cluster assignment were robust, achieving high mean accuracies (SBT-based: 0.89; ÖMPSQ-short-based: 0.87). The net benefit is superior throughout a range of threshold probabilities compared with a “treat-all” strategy.

**Conclusion.:**

A novel framework was developed that integrates multimodal data to identify distinct subgroups differentiated by their physical and psychosocial characteristics in a population-based cohort, demonstrating potential for advancing personalized care.

Effective treatment for low back pain (LBP) necessitates a personalized approach owing to the diverse and often multifactorial risk factors associated with LBP.^1^ Targeted treatments based on risk stratification of patients with LBP can offer improved outcomes and optimization of resources.^2^ In this regard, screening instruments such as Örebro Musculoskeletal Pain Screening Questionnaire (ÖMPSQ) short,^3^ STarT Back Tool (SBT)^4^ were developed to stratify the patients with LBP into risk groups. While these easy-to-use and clinically validated instruments are promising for identifying work absenteeism and future functional ability,^5–7^ their predictive performance remains limited, necessitating the inclusion of a broader range of factors including nonmodifiable factors.^5,7^

Emerging research underscores the association between magnetic resonance imaging (MRI)—derived findings and LBP highlighting the potential of imaging-based biomarkers.^8–10^ To this effect, several deep learning (DL) based frameworks have been developed for the analysis of lumbar spine MRI to extract imaging-based biomarkers related to disc degeneration^11–14^ (DD) and facet tropism^15^ (FT) which are found to have associations with LBP.^16,17^ These objectively quantifiable biomarkers offer a complementary perspective to self-reported questionnaires, enabling a more granular risk stratification.

While screening questionnaires such as the ÖMPSQ-short and SBT offer a straightforward approach to stratify and manage patients with LBP in primary care settings, they do not account for underlying physiological changes. Conversely, imaging biomarkers, despite their objectivity, do not capture the psychosocial dimensions of a patient’s risk profile. Employing either method in isolation may consequently lead to suboptimal outcomes in LBP management.^18^

To address current limitations in LBP patient stratification, this study introduces a novel, unsupervised framework. On the basis of the available literature, this is the first study to integrate DL-derived lumbar spine MRI biomarkers with demographic, lifestyle, and psychosocial data from the aforementioned questionnaires. The study hypothesized that this multimodal integration would identify clinically meaningful subgroups not discernible through methods relying solely on questionnaires or imaging. The clinical utility of this stratified approach was subsequently validated, demonstrating a superior net benefit over a “treat-all” strategy.

## MATERIALS AND METHODS

### Data Curation

This study utilized retrospective and cross-sectional data from the Northern Finland Birth Cohort^19,20^ (NFBC1966). Data was curated on demographic factors: sex and body mass index (BMI), lifestyle habits: smoking and psychosocial characteristics: SBT and ÖMSPQ-short questionnaires relevant to LBP.^21^ The study also used T2-weighted (T2w) lumbar spine MRIs of the participants acquired when they were 46 years old. Trained nurses conducted clinical examinations, and participants provided self-reported data for lifestyle and psychosocial characteristics through questionnaires. All the participants provided informed consent, and the imaging adhered to the Declaration of Helsinki, approved by the Northern Ostrobothnia Hospital District, Ethics Committee, 94/2011 (12.12.2011), Figure 1 illustrates the inclusion and exclusion criteria.

**Figure 1:**
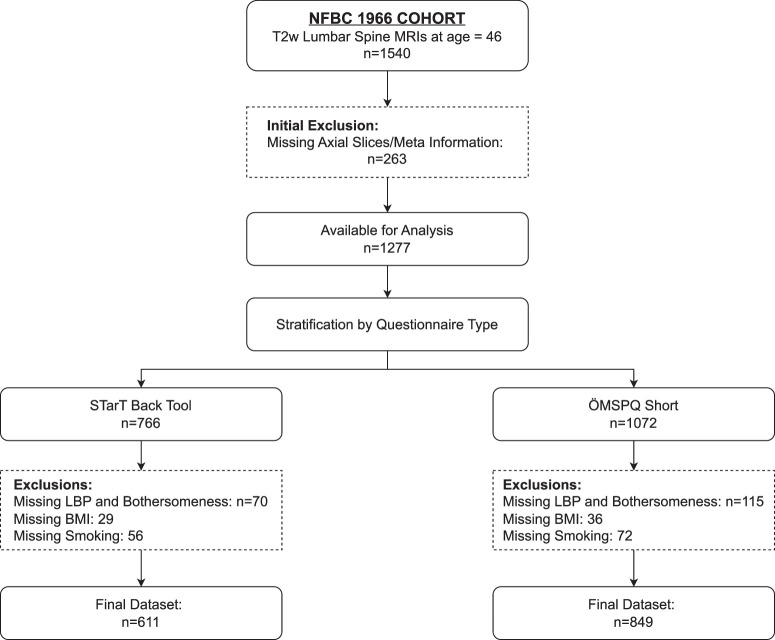
Flowchart illustrating the inclusion and exclusion criteria for participants from the Northern Finland Birth Cohort 1966. Participants were initially excluded if axial slices, or meta-information required for extracting imaging-based biomarkers. Further exclusions were made from missing self-reported data on low back pain frequency and bothersomeness, as well as smoking status. In addition, participants with missing body mass index (BMI) data were excluded.

Sex (male and female) was based on birth records. BMI (kg/m^2^) was assessed as part of the clinical examination, including participants’ height and weight measurements. For smoking status, participants were categorized into three groups based on their self-reported responses: current smokers, former smokers, and nonsmokers.^22^ LBP prevalence was determined using a two-part question. Participants first responded “Yes/No” to the question, “Have you had any aches or pain in your lower back within the last 12 months?” If they answered “Yes,” they then reported the frequency with “How often have you had aches or pains during the last 12 months?”, selecting from options including 1 to 7 days, 8 to 30 days, >30 days but not daily, and daily. Participants also rated pain-related overall bothersomeness at night, during leisure time and at work on a numerical scale from 0 to 10, where 10 indicated extremely severe or bothersome.

Psychosocial characteristics relevant to LBP experienced within the preceding year were assessed using clinically established instruments: SBT and ÖMPSQ-short. SBT, a nine-item questionnaire, identifies prognostic indicators for persistent disabling LBP to guide treatment.^5^ A 10-question short form of the ÖMPSQ was used to identify individuals at risk of long-term disability. Table 1 outlines a detailed description of the variables. Table 2 outlines the baseline characteristics of the study groups. Feature variable distributions (Supplementary Figures 1 and 2, Supplemental Digital Content 1, http://links.lww.com/BRS/C924), correlation heatmaps (Supplementary Figures 3 and 4, Supplemental Digital Content 1, http://links.lww.com/BRS/C924) and T2w MRI scan parameters are provided in the supplementary material, Supplemental Digital Content 1, http://links.lww.com/BRS/C924.

**TABLE 1:** Data Dictionary Outlining Participant Characteristics, Deep Learning Model Predictions, and Self-Reported Measures, Along With Their Respective Acquisition Methods and Scales

Demographic		
Variable	Acquisition	Value
Sex	Birth records	0 – Male; 1 - Female
BMI	Recorded at the time of the visit	Continuous
Deep learning model predictions		
mdl34	DL model prediction of Pfirrmann grade at L3/4	Pfirrmann grades 2, 3, 4, 5
mdl45	DL model prediction of Pfirrmann grade at L4/5	Pfirrmann grades 2, 3, 4, 5
mdl5s1	DL model prediction of Pfirrmann grade at L5/S1	Pfirrmann grades 2, 3, 4, 5
ft_l34	DL model prediction of Facet Tropism at L3-L4	Angle in degrees
ft_l45	DL model prediction of Facet Tropism at L4-L5	Angle in degrees
ft_l5s1	DL model prediction of Facet Tropism at L5-S1	Angle in degrees
Self-reported data		
Smoking	Self-reported questionnaire from an individual	0 – Nonsmoker 1 – Former smoker 2 – Current smoker
lbp_frequency	Self-reported on a questionnaire from an individual	0 – Never 1 – 1–7 d 2 – 8–30 d 3 – >30 d 4 – Daily
Bothersomeness	Self-reported on the questionnaire from the patient	Scale 1 to 10, where 10 is worse
Derived		
Risk_level	Computed from STarT Back Tool and ÖMPSQ-short questionnaire	0 – Low risk 1 – Medium risk 2 – High risk

BMI indicates body mass index; DL, deep learning; ÖMPSQ, Örebro musculoskeletal pain screening questionnaire.

**TABLE 2:** Baseline Characteristics of Individuals in the STarT Back Tool (SBT) and Örebro Musculoskeletal Pain Screening Questionnaire Short (ÖMPSQ Short) Study Groups

Feature	SBT		ÖMPSQ Short	
Sex	Male: 298 (48.7) Female: 313 (51.2)		Male: 384 (45.2) Female: 465 (54.7)	
BMI	Underweight: 3 (0.004) Healthy weight: 227 (37.1) Overweight: 271 (44.3) Obese: 110 (18)		Underweight: 2 (0.002) Healthy weight: 335 (39.4) Overweight: 359 (42.2) Obese: 153 (18.02)	
Disc degeneration	L3/4	Grade 2: 275(45)	L3/4	Grade 2: 395 (46.52)
		Grade 3: 259(42.3)		Grade 3: 366 (43.10)
		Grade 4: 64(10.4)		Grade 4: 72 (8.48)
		Grade 5: 13(2.12)		Grade 5: 16 (1.88)
	L4/5	Grade 2: 113 (18.49)	L4/5	Grade 2: 175 (20.61)
		Grade 3: 274 (44.84)		Grade 3: 397(46.76)
		Grade 4: 176 (28.80)		Grade 4: 226 (26.61)
		Grade 5: 48 (7.85)		Grade 5: 51 (6.00)
	L5/S1	Grade 2: 107 (17.51)	L5/S1	Grade 2: 179 (21.08)
		Grade 3: 191 (31.26)		Grade 3: 272 (32.03)
		Grade 4: 208 (34.04)		Grade 4: 266 (31.33)
		Grade 5: 105 (17.18)		Grade 5: 132 (15.54)
Facet tropism	L3/4	Normal: 517 (84.61)	L3/4	Normal: 714 (84.09)
		Moderate: 70 (11.45)		Moderate: 102 (10.12)
		Severe: 24 (3.92)		Severe: 33 (3.8)
	L4/5	Normal: 468 (76.59)	L4/5	Normal: 660 (77.73)
		Moderate: 98 (0.16)		Moderate: 121 (14.25)
		Severe: 45 (7.36)		Severe: 68 (8.00)
	L5/S1	Normal: 454 (74.30)	L5/S1	Normal: 637 (75.02)
		Moderate: 110 (18.00)		Moderate: 152 (17.90)
		Severe: 47 (7.69)		Severe: 60 (7.06)
Smoking	Nonsmokers: 307 (50.24) Former smokers: 186 (30.44) Current smokers: 118 (19.31)		Nonsmokers: 452 (53.23) Former smokers: 243 (28.62) Current smokers: 154 (18.13)	
Psychosocial risk level	Low: 542 (88.70) Medium: 58 (9.49) High: 11 (1.80)		Low: 716 (84.33) Medium: 78 (9.18) High: 55 (6.47)	
LBP frequency	Never: 106 (17.34) 1–7 d: 103 (16.85) 8–30 d: 155 (25.36) >30 d (not daily): 169 (27.65) Daily: 78 (12.76)		Never: 250 (29.44) 1–7 d: 148 (17.43) 8–30 d: 192 (22.61) >30 d (not daily): 182 (21.43) Daily: 77 (9.06)	
Bothersomeness	Low: 289 (47.29) Medium: 124 (20.29) High: 198 (32.40)		Low: 489 (57.59) Medium: 143 (16.84) High: 217 (25.5)	

The table presents a comparison of imaging biomarkers, demographic, clinical, and lifestyle factors. Values are presented as a number (percentage).

### Data Preprocessing

Imaging biomarkers were extracted from the MRI scans using predictions from a DL model.^14,15^ T2w mid-sagittal images were used to predict the Pfirrmann grades^23^ (a measure of DD) for lumbar levels L1/2 to L5/S1. In addition, the angle between facet joints was obtained from T2w axial images for lumbar levels L3/4 to L5/S1 to assess FT. Consequently, only Pfirrmann grades for lumbar levels L3/4 to L5/S1 were included in the final data set, as axial imaging was unavailable for levels L1/2 and L2/3.

Participant psychosocial risk was quantified by a single numerical score, calculated using the methodology outlined by Simula *et al*.^24^ Categorical variables were defined with the following encoding: psychosocial risk (0=low, 1=medium, 2=high); smoking status (0=nonsmokers, 1=former smokers, 2=current smokers); sex (0=male, 1=female); LBP frequency (0=no pain, 1=1 to 7 days, 2=8 to 30 days, 3=>30 days but not daily, 4=daily) and bothersomeness was encoded as (0 to 3=low, 4 to 5=moderate, 6 to 10=high). A composite category, “Frequent Pain” was defined by combining LBP frequencies of “Greater than 30 days but not daily” and “Daily.” To facilitate reporting and analysis, several continuous variables were binned: DD was categorized as mild (grade 2), moderate (grade 3), or severe (grades 4 to 5) degeneration; FT was classified as normal (0 to 10 degrees), moderately accentuated (10 to 15 degrees), or severely accentuated (>15 degrees); and BMI categories followed established World Health Organization guidelines.

### Unsupervised Patient Stratification

#### Data Scaling

Before K-Means clustering, the variables were standardized to a common scale. This is necessary as K-Means clustering is highly sensitive to scale differences due to its reliance on the Euclidean distance metric.^25^ The data set included continuous variables (*e.g.*, BMI and FT) and ordinal numeric variables with heterogeneous scales. To address this, the data were initially scaled using RobustScaler from the scikit-learn library (version 1.7.0), a method effective in mitigating outlier influence. Following scaling, dimensionality reduction was performed using Uniform Manifold Approximation and Projection (UMAP),^26^ a nonlinear technique that preserves both local and global data structures in lower-dimensional representations.

#### K-Means Clustering

K-means clustering was performed separately for each psychosocial risk tool (ÖMPSQ-short and SBT). Each analysis integrated imaging biomarkers (DD and FT), smoking status, BMI, and sex. Unlike conventional single-pass clustering, a robust clustering methodology was used inspired by the five-fold cross-validation paradigm typically used in supervised learning. A primary challenge with applying such a technique to unsupervised clustering lies in the arbitrary nature of cluster labels generated in each independent run.

**Figure 2:**
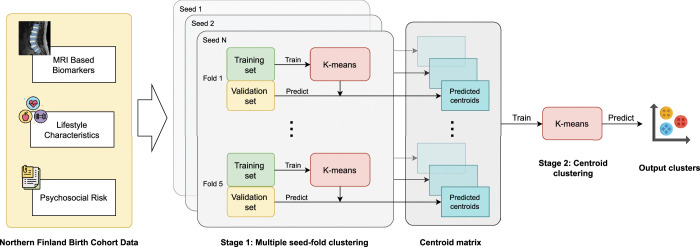
Overall pipeline of the five-fold cross-validation of the K-means clustering algorithm using a centroid matrix. The data set for the clustering analysis was curated from the Northern Finland Birth Cohort 1966. This data set comprises demographic, lifestyle, deep learning (DL) based imaging biomarkers and psychosocial characteristics obtained from clinically validated instruments, including the STarT Back Tool (SBT) and Örebro Musculoskeletal Pain Screening Questionnaire (ÖMSPQ) short questionnaires.

To circumvent this issue, rather than storing the arbitrary cluster labels directly, the cluster centroids corresponding to each UMAP-transformed validation set were instead recorded within each fold. Consequently, upon completion of the five-fold process, a matrix of cluster centroids was obtained for every UMAP-transformed data point in the data set. A second pass was then applied to this centroid matrix to derive the final, consistent cluster labels, which were subsequently assigned to their respective data points. The optimal hyperparameters for UMAP, specifically the number of UMAP components, the number of UMAP neighbors, and the minimum distance, along with the value of *k* for the clusters, were selected using a grid search approach. The best-performing values were then used for the final clustering. Figure 2 illustrates the overall clustering algorithm. The goodness of the formed clusters was measured using the Silhouette score, Davies-Bouldin Index, and Calinski-Harabasz score, all computed with libraries from scikit-learn (version 1.7.0).

#### Cluster Characterization and Predictive Modeling

To characterize each cluster, the within-cluster distribution of LBP Frequency and bothersomeness was first assessed. For each cluster, the odds ratio (OR) of its specific features, adjusted for the confounders (sex, BMI, and smoking) was computed with both LBP Frequency and bothersomeness as target variables. Subsequently, a multiclass logistic regression model was fit using a five-fold cross-validation approach, with the derived cluster labels as targets. The data set was scaled using RobustScaler before fitting the logistic regression model, which was implemented using the statsmodels library (version 0.14.5).

Model performance was evaluated using precision-recall curves and receiver operating characteristic (ROC) curves. To account for uncertainty, these performance metrics were assessed across 1000 bootstrap samples. Finally, decision curve analysis (DCA)^27^ was performed to evaluate the clinical net benefit of the model. Net benefit quantifies the clinical value of using the prediction model by rewarding correct cluster assignments and penalizing incorrect ones, moving beyond simple accuracy. Clinical utility is then demonstrated when the model’s net benefit curve on the DCA plot surpasses the “treat all” or “treat none” strategies across a range of clinical risk thresholds.

## RESULTS

### Emergence of Clusters and Characteristics

K-means clustering was performed on the curated data, which included imaging biomarkers, lifestyle characteristics, and psychosocial characteristics. The optimal clustering, achieved with k=3, utilized the following UMAP hyperparameters: seven components, 20 neighbors, and a minimum distance of 0.3. This configuration yielded superior cluster separation for the SBT compared with the ÖMPSQ-short, as evidenced by a higher Silhouette Score (0.78 *vs.* 0.63), a lower Davies-Bouldin Index (0.33 *vs.* 0.67), and a higher Calinski-Harabasz Score (4937.14 *vs.* 4223.61). Visualization of clusters (Supplementary Figures 5 and 6, Supplemental Digital Content 1, http://links.lww.com/BRS/C924), distribution of features for each cluster (Supplementary Figures 7-10, Supplemental Digital Content 1, http://links.lww.com/BRS/C924; Supplementary Tables 1 and 2, Supplemental Digital Content 1, http://links.lww.com/BRS/C924) are provided in the supplementary material, Supplemental Digital Content 1, http://links.lww.com/BRS/C924.

### SBT Clusters

**Figure 3:**
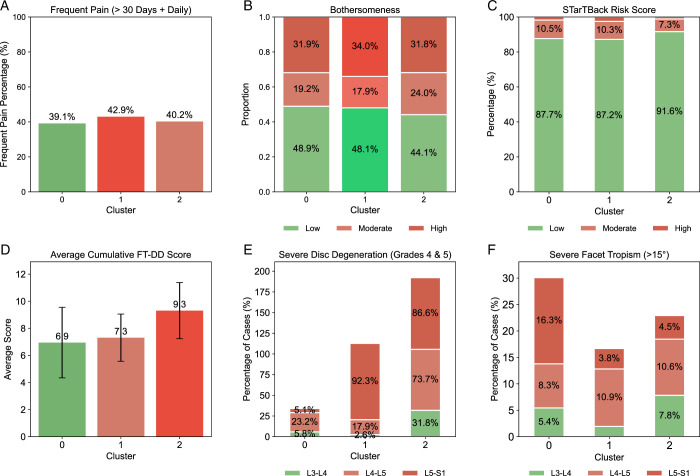
STarT Back Tool (SBT) cluster evaluation. (A) The bar chart shows the percentage of frequent pain (>30 d + daily) across the three identified clusters. (B) Proportion of bothersomeness (low, moderate, and high) within each cluster. (C) SBT Risk Score distribution (low, moderate, and high) for each cluster. (D) Average cumulative FT-DD Score for each cluster, including error bars. (E) Percentage of cases with severe disc degeneration (grades 4 and 5) at different spinal levels (L3/4, L4/5, L5/S1) across the clusters. (F) Percentage of cases with severe facet tropism (>15°) at different spinal levels (L3/4, L4/5, L5/S1) across the clusters.

The prevalence of frequent pain and pain bothersomeness was evaluated across the SBT clusters. Cluster 1 exhibited the highest proportion of frequent pain (42.9%) (Fig. 3A), closely followed by Cluster 2 (40.2%). Pain-driven bothersomeness was also higher in Cluster 1 (34.0%) compared with Cluster 2 (31.8%) (Fig. 3B). Compared with Clusters 1 and 2, Cluster 0 had the lowest frequent pain (39.1%) and DD at L5/S1 (5.1%) along with a lower cumulative FT and DD score (6.9).

Severe DD was notably more prevalent in Cluster 2 (Fig. 3E), particularly at L5/S1 (86.6%) and L4/5 (73.7%), when compared with Cluster 1. The cumulative scores for FT and DD severity were also highest in Cluster 2 (9.3), followed by Cluster 1 (7.3) (Fig. 3D). Interestingly, Cluster 1, which had a higher prevalence of frequent pain, also displayed a slightly higher SBT risk score (2.5%) (Fig. 3C) compared with Cluster 2(1.1%), but the percentage was nearly the same when compared with cluster 0 (2.8%). Notably, Cluster 0 with the lowest frequent pain shows more severe FT (>15°) than Clusters 1 and 2 (Fig. 3F).

**Figure 4:**
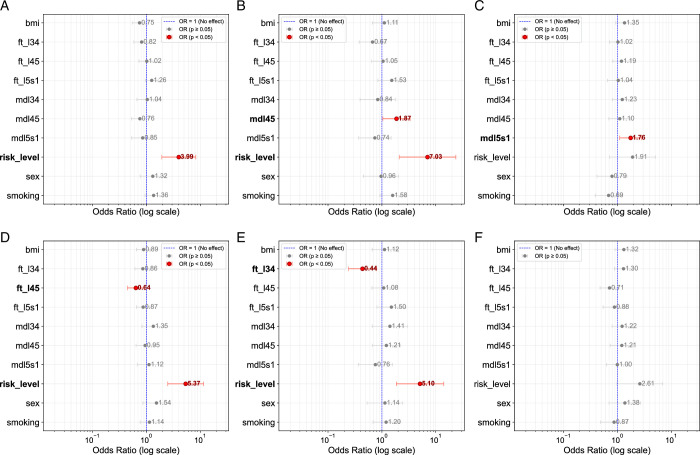
STarT back tool odds ratios per cluster and target. The figure presents odds ratios (log scale) for cluster features across three clusters (Cluster 0, Cluster 1, and Cluster 2) with respect to two target variables: Frequent pain (top row) and bothersomeness (bottom row). Subplots (A), (B), (C) represent the odds ratio of features with frequent pain as the target variable for clusters 0, 1 and 2, respectively. Subplots (D), (E), (F) represent odds ratios of features with bothersomeness as the target variable for clusters 0, 1, and 2. Red markers indicate statistically significant odds ratios (*P *< 0.05) and no effect (OR=1) is plotted with a dashed blue line.

Further analysis revealed distinct characteristics for frequent pain and pain-driven bothersomeness within each cluster. In Cluster 1, DD at L4/5 (OR=1.87; *P*<0.05) combined with psychosocial risk (OR=7.03; *P *< 0.05) significantly characterized frequent pain (Fig. 4B). In addition, the psychosocial risk score continued to be significant (OR=5.10; *P *< 0.05) along with the protective feature FT at L3/4 (OR=0.44; *P *< 0.05) for pain-driven bothersomeness in Cluster 1 (Fig. 4E). In contrast, only DD at L5/S1 (OR=1.76; *P *< 0.05) significantly characterized Cluster 2 for frequent pain (Fig. 4C), with neither the psychosocial risk score nor DD or FT-related features showing significance for pain-driven bothersomeness in this cluster. However, the psychosocial risk score nearly exceeded statistical significance in both models. Figure 4A–C presents a forest plot of the significant cluster features, indicating their respective odds ratios for frequent pain for clusters 0, 1, and 2. Figure 4D–F represents the pain-related bothersomeness for the same clusters.

### ÖMPSQ-Short Clusters

**Figure 5:**
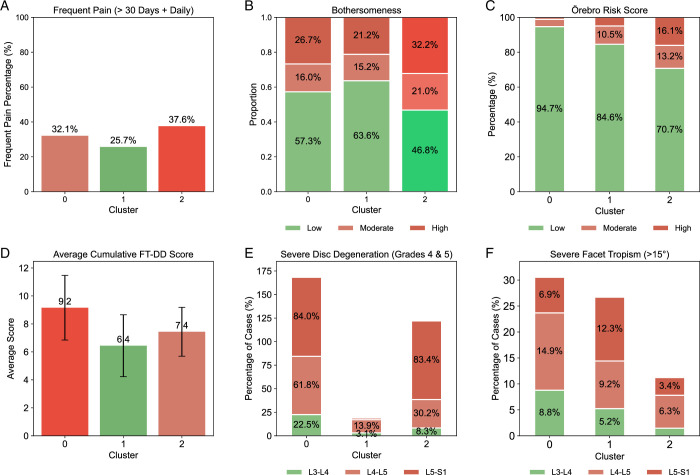
Örebro musculoskeletal pain screening questionnaire (ÖMPSQ) short cluster evaluation. (A) The bar chart shows the percentage of frequent pain (>30 d + Daily) across the three identified clusters. (B) Proportion of bothersomeness (low, moderate, and high) within each cluster. (C) ÖMSPQ Short Risk Score distribution (low, moderate, and high) for each cluster. (D) Average cumulative FT-DD Score for each cluster, including error bars. (E) Percentage of cases with severe disc degeneration (grades 4 and 5) at different spinal levels (L3/4, L4/5, L5/S1) across the clusters. (F) Percentage of cases with severe facet tropism (>15°) at different spinal levels (L3/4, L4/5, L5/S1) across the clusters.

The cluster characteristics derived from the ÖMPSQ-short analysis largely mirrored those from the SBT. Cluster 2 displayed the highest prevalence of frequent pain (37.6%) (Fig. 5A), followed by Cluster 0 (32.1%). Similarly, pain-driven bothersomeness was greater in Cluster 2 (32.2%) compared with Cluster 0 (26.7%) (Fig. 5B). Cluster 1 contrasts with Cluster 0 and 2 with lower Frequent Pain (25.7%) and pain-driven bothersomeness (21.2%).

Cluster 2 also exhibited a significantly higher ÖMPSQ-short score (16.1%) when compared with Cluster 0 (<5%) (Fig. 5C). Cluster 0 showed a higher prevalence of both the cumulative FT and DD severity scores (9.2) (Fig. 5D). This cluster was also characterized by severe DD, particularly at L5/S1 (84.0%) and L4/L5 (61.8%) (Fig. 5E), and higher FT prevalence at L5/S1 (6.9%) and L4/L5 (14.9%) (Fig. 5F).

**Figure 6:**
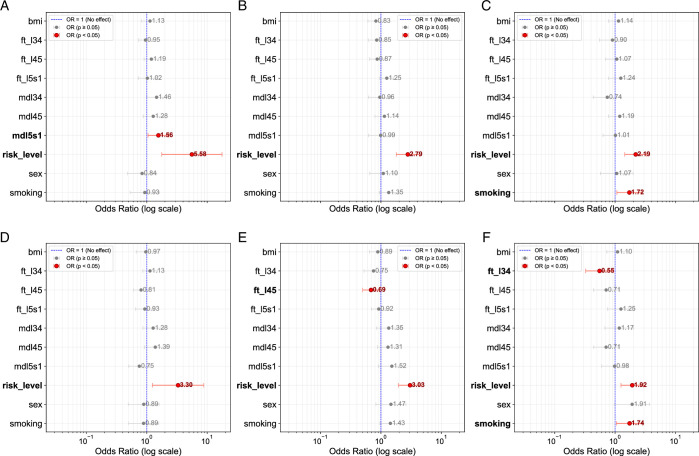
Örebro musculoskeletal pain screening questionnaire (ÖMPSQ) short odds ratios per cluster and target. The figure presents odds ratios (log scale) for cluster features across three clusters (Cluster 0, Cluster 1, and Cluster 2) with respect to two target variables: frequent pain (top row) and bothersomeness (bottom row). Subplots A, B, C represent the odds ratios of features with Frequent Pain as the target variable for clusters 0, 1 and 2, respectively. Subplots D, E, and F represent the odds ratios of features with Bothersomeness as the target variable for clusters 0, 1, and 2. Red markers indicate statistically significant odds ratios (*P*<0.05) and no effect (OR=1) is plotted with a dashed blue line.

Further examination of within-cluster characteristics for frequent pain and pain-driven bothersomeness revealed distinct patterns. Cluster 2, which presented with a higher psychosocial risk score, was significantly characterized by both psychosocial risk score (OR=2.19; *P *< 0.05) and smoking (OR=1.72; *P *< 0.05) for frequent pain (Fig. 6C). In contrast, Cluster 0, predominantly marked by higher DD and FT, showed DD at L5/S1 (OR=1.56; *P *< 0.05) and psychosocial risk score (OR=5.58; *P *< 0.05) as statistically significant for frequent pain (Fig. 6A).

Regarding pain-driven bothersomeness, Cluster 2 was primarily driven by smoking (OR=1.74; *P *< 0.05) and psychosocial risk (OR=1.92; *P *< 0.05), with FT at L3/4 serving as a protective feature (OR=0.55 ; *P *< 0.05) (Fig. 6F). Conversely, Cluster 0 demonstrated psychosocial risk (OR=3.30; *P *< 0.05) as its sole significant characteristic for pain-driven bothersomeness (Fig. 6D). Figure 6–C presents a forest plot of the significant cluster features, indicating their respective odds ratios for frequent pain for ÖMPSQ-short clusters 0, 1, and 2. Figure 6D–F represents the pain-related bothersomeness for the same clusters.

### Predictive Modeling Supports Cluster Robustness

**Figure 7:**
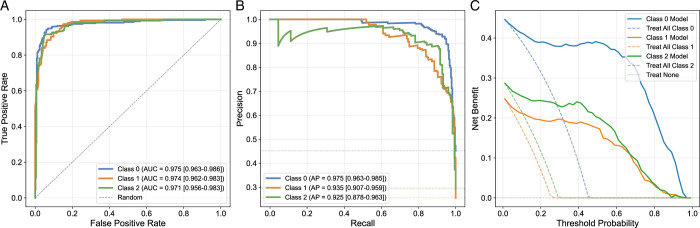
STarT Back Tool (SBT) logistic regression model evaluation. The figure displays various performance metrics for the SBT model across three classes (Cluster 0, Cluster 1, and Cluster 2), with uncertainty calculated through 1000 bootstrap sampling runs. (A) Receiver Operating Characteristic plot with confidence bands for each class. (B) Precision-Recall Curves with confidence bands for each class. (C) Net Benefit with CI, comparing the model’s performance against “Treat All” and “Treat None” strategies for each class.

**Figure 8:**
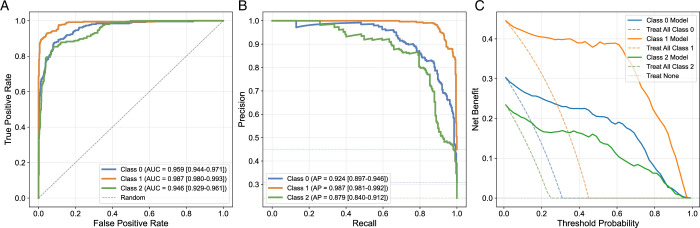
Örebro musculoskeletal pain screening questionnaire (ÖMPSQ) short logistic regression model evaluation. The figure displays various performance metrics for the ÖMPSQ Short model across three classes (Cluster 0, Cluster 1, and Cluster 2), with uncertainty calculated through 1000 bootstrap sampling runs. (A) Receiver operating characteristic plot with confidence bands for each class. (B) Precision-recall curves with confidence bands for each class. (C) Net Benefit with CIs, comparing the model’s performance against “Treat All” and “Treat None” strategies for each class.

The predictive model, which stratified participants based on their demographic, lifestyle, psychosocial scores, and imaging biomarkers for DD and FT, demonstrated consistent performance across all cluster labels. The SBT-based model achieved a mean accuracy of 0.89±0.01, while the ÖMPSQ-short model performed similarly with a mean accuracy of 0.87±0.02 from five-fold cross-validation. These trained models significantly outperformed models with randomly shuffled cluster labels, which yielded mean accuracies of 0.42±0.02 for the SBT-based model and 0.43±0.01 for the ÖMPSQ-short model, confirming that the model was better than random assignment. Furthermore, Figures 7 (for the SBT model) and 8 (for the ÖMPSQ-short) visually demonstrate the robust performance of the models. They illustrate the ROC (Figs. 7A, 8A) and Precision-Recall curves (Figs. 7B, 8B), both accompanied by 95% CIs.

### Net Benefit of Stratified Risk Assessment

Net benefit analysis (Figs. 7C and 8C) for both the SBT and ÖMPSQ-short models consistently demonstrated that a stratified risk-based approach based on cluster assignments offered greater utility compared with a “treat-all” strategy. The highest net benefit was observed in the cluster characterized by low-frequency pain, lower DD, and a lower cumulative FT-DD score across both models.

While other clusters characterized by higher psychosocial risk and higher DD showed moderate net benefit, their performance remained significantly superior to the “treat-all” strategy. More specifically, the cluster exhibiting a higher psychosocial risk profile consistently demonstrated a slightly lower net benefit compared with the cluster characterized by higher cumulative DD and severe DD across different threshold probabilities for both models. For example, at a threshold probability of 0.4, the SBT model’s Cluster 2 (severe DD) yielded a net benefit of 0.25, while Cluster 1 (high psychosocial risk) showed a net benefit of 0.19 (Fig. 7C). Similarly, for the ÖMPSQ-short model at the same 0.4 threshold, Cluster 0 (highest DD/FT-DD) yielded a net benefit of 0.22, marginally outperforming Cluster 2 (highest psychosocial risk) at 0.18 (Fig. 8C). As the threshold probability increases, both models exhibit a steep decline in net benefit; however, they consistently outperform the “treat-all” strategy across the range of threshold probabilities.

## DISCUSSION

This study introduced a robust unsupervised patient stratification approach for individuals experiencing LBP within the NFBC1966 cohort. Diverging from conventional primary care stratification methods that often rely solely on questionnaires, a novel framework integrated demographic, lifestyle, and psychosocial factors with advanced DL-based imaging biomarkers derived from T2w lumbar spine MRIs. This comprehensive approach stratified patients into three distinct subgroups, each exhibiting varied proportions of frequent pain and bothersomeness. Importantly, the goal of this multimodal stratification is to help clinicians determine treatment choices by better characterizing patient subgroups.

The integration of MRI-based imaging biomarkers with lifestyle, demographic, and clinical data (SBT, ÖMPSQ-short) in LBP patient stratification limits direct comparability with existing literature. Nevertheless, across clusters derived from both models, the cluster with the highest proportion of individuals reporting frequent pain was characterized by a complex interplay of psychosocial risk factors and degenerative morphology. The cluster with the second-highest pain prevalence was defined predominantly by degenerative morphology alone. These findings align with the biopsychosocial nature of LBP^28^ and highlight the importance of incorporating biological markers into risk stratification.

While the SBT model yielded better technical clustering scores, the ÖMPSQ-short model produced subgroups with more pronounced differences in both Frequent Pain and psychosocial risk scores. Notably, the ÖMPSQ-short risk score was significantly associated with pain-related bothersomeness and consistent with prior findings.^24,29^ Ultimately, the significant odds ratios associated with the imaging biomarkers and the net benefit of both models underscore the potential of advanced imaging data for improving patient stratification.

Despite these strengths, this study has several limitations. It is limited by its cross-sectional design, which prevents causal inferences and the observation of longitudinal changes. The study is also limited by its reliance on disc degeneration (DD) and facet tropism (FT) as imaging biomarkers; incorporating a broader range of imaging (disc herniation, spinal stenosis, paraspinal muscles) could potentially enhance patient stratification. As for the incorporation of nonimaging biomarkers, a recent UK Biobank study with over 532,000 participants showed that biomarkers such as immunoassays, brain imaging, and polygenetic risk scores predict painful medical conditions like ankylosing spondylitis, but that the presence and impact of persistent pain, including back pain, are solely determined by psychosocial factors.^30^ The reliance on self-reported data introduces potential recall and social desirability biases^31^ and measurement bias from the clinical examinations^32^ should also be acknowledged. Finally, the study’s findings require external validation to ensure generalizability, and future research should explore tailored intervention strategies for the identified clusters.

## CONCLUSION

This study has established a foundational approach to comprehensively combine patient-specific imaging biomarkers with established clinical questionnaires (SBT and ÖMPSQ-short), demographics and lifestyle factors. It has been shown that the proposed method integrates these diverse data modalities, leading to the identification of robust and distinct subgroups for frequent LBP and bothersomeness. The consistent performance and improved net benefit of the predictive models, when compared with a “treat-all” strategy, underscores their potential for advancing personalized care for LBP. Ultimately, these models are intended to allow future prospective studies to validate the clinical relevance of these subgroups by measuring meaningful outcomes like health-related quality of life, functional, and work ability.

Key Points

- We introduced a robust, multimodal unsupervised framework for low back pain patient stratification that combines psychosocial, lifestyle, demographic, and MRI data.
- We demonstrated that this approach identifies distinct subgroups, each with a unique profile characterized by different weightings of psychosocial risk and disc degeneration in a population-based cohort.
- We validated the clinical utility of our stratification model by showing it provides superior net benefit compared with a “treat-all” strategy.

## Supplementary Material

SUPPLEMENTARY MATERIAL
