# Supplementary material for: An Unsupervised Learning Approach for Multimodal Low Back Pain Stratification
Source: Spine (Phila Pa 1976). 2025 Dec 22;51(8):582–92. doi: 10.1097/BRS.0000000000005593 (PMC13011946; doi:10.1097/BRS.0000000000005593)
Supplement: SUPPLEMENTARY MATERIAL [file brs-51-582-s001.pdf]

Supplementary Material

NFBC MRI Imaging and Parameters:

All imaging, conducted between 2011 and 2015, utilized a 1.5 Tesla GE Signa HDxt scanner (General Electric, Milwaukee, WI, USA). We acquired T2-weighted fast-recovery fast spin-echo images in both sagittal and axial orientations. For sagittal scans, the parameters were: TR/effTE 3500/112 ms, pixel spacing 0.5 mm, slice thickness 3 mm, field of view 280x280 mm, and 512x512 pixel resolution. Axial imaging, which was limited to the L3/4 through L5/S1 vertebral levels, employed slightly different parameters: TR/effTE 3600/118 ms, pixel spacing 0.3 mm, slice thickness 4 mm, field of view 180x180 mm, and 512x512 pixel resolution.

SBT and ÖMPSQ Short Questionnaires

SBT questionnaire includes eight dichotomous statements (Likert-type scale: 0 = disagree, 1 = agree) and one five-point bothersomeness item, scored as 0 = not at all to moderately bothersome and 1 = much to very much bothersome, resulting in a total sum score ranging from 0 to 9[1]. Higher scores indicate a higher risk. Overall, SBT captures pain-related bothersomeness, referred leg pain, comorbid pain, disability (two questions), catastrophizing, fear, and anxiety, and depressive symptoms. SBT has been validated in Finnish[2].

ÖMPSQ Short was developed to identify individuals at risk of long-term work disability[3]. Ten items scaled 0 to 10 inquire about (1) the duration of pain(s), (2) pain rating, (3) the ability to do light work, (4) the ability to sleep at night, (5) anxiety feelings, (6) depressed feelings, (7) the perceived risk of pain becoming chronic, (8) self-estimate of return to work and (9–10) fear-avoidance beliefs[1]. The total sum score ranges between 0 and 100 points, with higher values reflecting a higher risk. The original version of ÖMPSQ has been validated in Finnish[4].

SBT and ÖMPSQ Short Psychosocial Risk Characterization

Participant's psychosocial risk was categorized into three risk groups (low, medium and high) for both SBT and ÖMPSQ Short based questionnaires[1]. The categorical variables were defined with the following encoding: psychosocial risk (0 = Low [SBT: total sum score ≤ 3 points; ÖMSPQ-short: 0–39 points], 1 = Medium [SBT: total sum score ≥ 4 points and psychosocial subscale score ≤ 3 points; ÖMSPQ-short: 40–49 points], 2 = High [SBT: both total sum score and psychosocial subscale score ≥ 4 points; ÖMSPQ-short: 50–100 points]).

SBT and ÖMPSQ Short Feature Distribution

Frequency distribution plots were generated for both the STarT Back Tool (SBT, **Supplementary Figure 1**) and ÖMPSQ Short (**Supplementary Figure 2**) for each feature within the dataset. These features, along with their numerical encodings where applicable, include:

- **Sex**: Encoded as 0 (Male) and 1 (Female).
- **BMI**: A continuous variable representing Body Mass Index.
- **Disc Degeneration**: Categorized as 2 (Mild), 3 (Moderate), and 4-5 (Severe).
- **Facet Tropism**: A continuous variable representing the angle between facet joints.
- **Smoking**: Encoded as 0 (Non-Smoker), 1 (Former Smoker), and 2 (Current Smoker).
- **Risk Level**: Categorized as 0 (Low Risk), 1 (Medium Risk), and 2 (High Risk).
- **Low Back Pain Frequency**: Encoded as 0 (Never), 1 (1 to 7 days), 2 (8 to 30 days), 3 (Greater than 30 days), and 4 (Daily).
- **Pain-Related Bothersomeness**: Ranging from 0 (Low) to 10 (High).

SBT and ÖMPSQ Short Feature Correlation:

A Pearson correlation analysis was conducted, and a heatmap was generated to visualize statistically significant variables ($p < 0.05$). A higher correlation extent (≥0.20) was observed for disc degeneration at the L3/4, L4/5, and L5/S1 levels. In contrast, other statistically significant variables exhibited lower correlation strengths (<0.10). Despite the observed higher correlations among disc degeneration at different levels, these spinal segments are considered anatomically independent. Consequently, these features were included as independent variables in the subsequent clustering analysis. **Supplementary Figures 3 and 4** illustrate the heatmaps for SBT and ÖMPSQ Short respectively.

Cluster Visualization of SBT and ÖMPSQ Short

To visualize the results of the K-means clustering, Plotly Express (Version 6.0.0) was used. The scaled dataset was first transformed into a three-dimensional representation using UMAP (Uniform Manifold Approximation and Projection) to reduce dimensionality. Subsequently, all three UMAP components were plotted, with the distinct cluster assignments highlighted by

color (hue). **Supplementary Figures 5 and 6** illustrates the cluster representations for SBT and ÖMPSQ Short respectively

Within Cluster Proportions SBT and ÖMPSQ Short

A stacked bar plot was utilized to illustrate the characteristics of within-cluster membership for each feature. **Supplementary Figures 7 and 8**, respectively, present the within-cluster feature proportions for the SBT and ÖMPSQ Short instruments.

Radar plots of SBT and ÖMPSQ Short Clusters

Radar plots were employed to visually illustrate the mean feature differences across the identified clusters. **Supplementary Figures 9 and 10**, respectively, display these feature distributions across clusters for the SBT and ÖMPSQ Short instruments, with a further breakdown by sex where applicable. While differences in feature profiles were observed between clusters, the radar plots for male and female subgroups within the same cluster demonstrated consistent profiles, suggesting that the underlying cluster characteristics are not markedly distinct across sexes. A key observation from ÖMPSQ Short plot is the co-occurrence of higher psychosocial risk with a greater extent of smoking.

Cluster Characteristics – SBT and ÖMPSQ Short

Cluster characteristics for both SBT and ÖMPSQ Short are presented in **Supplementary Table 1 and supplementary Table 2**

Supplementary Table1: Cluster characteristics based on SBT instrument for psychosocial risk.

Cluster	Characteristic	Distribution
0	Sex	Female: 161 (58.3%)<br>Male: 115 (41.7%)

	BMI	Healthy Weight: 92 (33.3%)<br>Obese: 55 (19.9%)<br>Overweight: 128 (46.4%)<br>Underweight: 1 (0.4%)
	Smoking	Current Smokers: 32 (11.6%)<br>Non-Smokers: 161 (58.3%)<br>Quitters: 83 (30.1%)
	Disc Degeneration	Grade 2: 306 (37.0%)<br>Grade 3: 428 (51.7%)<br>Grade 4: 83 (10.0%)<br>Grade 5: 11 (1.3%)
	Facet Tropism	Moderate: 70 (8.5%)<br>Normal: 709 (85.6%)<br>Severe: 49 (5.9%)
	Psychosocial Risk Level	High Risk: 5 (1.8%)<br>Low Risk: 242 (87.7%)<br>Medium Risk: 29 (10.5%)
	Low Back Pain Frequency	1-7 days: 46 (16.7%)<br>8-30 days: 69 (25.0%)<br>>30 days (not daily): 75 (27.2%)<br>Daily: 33 (12.0%)<br>No Back Pain: 53 (19.2%)
	Bothersomeness	Mild: 164 (59.4%)<br>Moderate: 70 (25.4%)<br>Severe: 42 (15.2%)
1	Sex	Female: 69 (44.2%)<br>Male: 87 (55.8%)
	BMI	Healthy Weight: 65 (41.7%)<br>Obese: 18 (11.5%)<br>Overweight: 71 (45.5%)<br>Underweight: 2 (1.3%)

	Smoking	Current Smokers: 73 (46.8%)<br>Non-Smokers: 35 (22.4%)<br>Quitters: 48 (30.8%)
	Disc Degeneration	Grade 2: 150 (32.1%)<br>Grade 3: 142 (30.3%)<br>Grade 4: 138 (29.5%)<br>Grade 5: 38 (8.1%)
	Facet Tropism	Moderate: 36 (7.7%)<br>Normal: 412 (88.0%)<br>Severe: 20 (4.3%)
	Psychosocial Risk Level	High Risk: 4 (2.6%)<br>Low Risk: 136 (87.2%)<br>Medium Risk: 16 (10.3%)
	Low Back Pain Frequency	1-7 days: 20 (12.8%)<br>8-30 days: 36 (23.1%)<br>>30 days (not daily): 46 (29.5%)<br>Daily: 21 (13.5%)<br>No Back Pain: 33 (21.2%)
	Bothersomeness	Mild: 85 (54.5%)<br>Moderate: 49 (31.4%)<br>Severe: 22 (14.1%)
	Sex	Female: 83 (46.4%)<br>Male: 96 (53.6%)
	BMI	Healthy Weight: 70 (39.1%)<br>Obese: 37 (20.7%)<br>Overweight: 72 (40.2%)
	Smoking	Current Smokers: 13 (7.3%)<br>Non-Smokers: 111 (62.0%)<br>Quitters: 55 (30.7%)
2	Disc Degeneration	Grade 2: 39 (7.3%)<br>Grade 3: 154 (28.7%)

		Grade 4: 227 (42.3%)<br>Grade 5: 117 (21.8%)
	Facet Tropism	Moderate: 50 (9.3%)<br>Normal: 460 (85.7%)<br>Severe: 27 (5.0%)
	Psychosocial Risk Level	High Risk: 2 (1.1%)<br>Low Risk: 164 (91.6%)<br>Medium Risk: 13 (7.3%)
	Low Back Pain Frequency	1-7 days: 37 (20.7%)<br>8-30 days: 50 (27.9%)<br>>30 days (not daily): 48 (26.8%)<br>Daily: 24 (13.4%)<br>No Back Pain: 20 (11.2%)
	Bothersomeness	Mild: 99 (55.3%)<br>Moderate: 49 (27.4%)<br>Severe: 31 (17.3%)

Supplementary Table2: Cluster characteristics based on ÖMPSQ Short instrument for psychosocial risk.

Cluster	Characteristic	Distribution
0	Sex	Female: 123 (46.9%)<br>Male: 139 (53.1%)
	BMI	Healthy Weight: 102 (38.9%)<br>Obese: 47 (17.9%)<br>Overweight: 113 (43.1%)
	Smoking	Current Smokers: 9 (3.4%)<br>Ex-Smokers: 63 (24.0%)<br>Non-Smokers: 190 (72.5%)
	Disc Degeneration	Grade 2: 92 (11.7%)<br>Grade 3: 253 (32.2%)<br>Grade 4: 311 (39.6%)<br>Grade 5: 130 (16.5%)
	Facet Tropism	Moderate: 77 (9.8%)<br>Normal: 655 (83.3%)<br>Severe: 54 (6.9%)
	Psychosocial Risk Level	High Risk: 3 (1.1%)<br>Low Risk: 248 (94.7%)<br>Medium Risk: 11 (4.2%)
	Low Back Pain Frequency	1-7 days: 55 (21.0%)<br>8-30 days: 61 (23.3%)<br>>30 days (not daily): 58 (22.1%)<br>Daily: 26 (9.9%)<br>No Back Pain: 62 (23.7%)
	Bothersomeness	Mild: 169 (64.5%)<br>Moderate: 60 (22.9%)<br>Severe: 33 (12.6%)

	Sex	Female: 243 (63.6%)<br>Male: 139 (36.4%)
	BMI	Healthy Weight: 142 (37.2%)<br>Obese: 72 (18.8%)<br>Overweight: 168 (44.0%)
	Smoking	Current Smokers: 37 (9.7%)<br>Ex-Smokers: 111 (29.1%)<br>Non-Smokers: 234 (61.3%)
	Disc Degeneration	Grade 2: 501 (43.7%)<br>Grade 3: 573 (50.0%)<br>Grade 4: 64 (5.6%)<br>Grade 5: 8 (0.7%)
	Facet Tropism	Moderate: 94 (8.2%)<br>Normal: 992 (86.6%)<br>Severe: 60 (5.2%)
	Psychosocial Risk Level	High Risk: 19 (5.0%)<br>Low Risk: 323 (84.6%)<br>Medium Risk: 40 (10.5%)
	Low Back Pain Frequency	1-7 days: 65 (17.0%)<br>8-30 days: 75 (19.6%)<br>>30 days (not daily): 73 (19.1%)<br>Daily: 25 (6.5%)<br>No Back Pain: 144 (37.7%)
1	Bothersomeness	Mild: 274 (71.7%)<br>Moderate: 73 (19.1%)<br>Severe: 35 (9.2%)
	Sex	Female: 99 (48.3%)<br>Male: 106 (51.7%)
2	BMI	Healthy Weight: 91 (44.4%)<br>Obese: 34 (16.6%)

		Overweight: 78 (38.0%)<br>Underweight: 2 (1.0%)
	Smoking	Current Smokers: 108 (52.7%)<br>Ex-Smokers: 69 (33.7%)<br>Non-Smokers: 28 (13.7%)
	Disc Degeneration	Grade 2: 156 (25.4%)<br>Grade 3: 209 (34.0%)<br>Grade 4: 189 (30.7%)<br>Grade 5: 61 (9.9%)
	Facet Tropism	Moderate: 46 (7.5%)<br>Normal: 551 (89.6%)<br>Severe: 18 (2.9%)
	Psychosocial Risk Level	High Risk: 33 (16.1%)<br>Low Risk: 145 (70.7%)<br>Medium Risk: 27 (13.2%)
	Low Back Pain Frequency	1-7 days: 28 (13.7%)<br>8-30 days: 56 (27.3%)<br>>30 days (not daily): 51 (24.9%)<br>Daily: 26 (12.7%)<br>No Back Pain: 44 (21.5%)
	Bothersomeness	Mild: 112 (54.6%)<br>Moderate: 63 (30.7%)<br>Severe: 30 (14.6%)

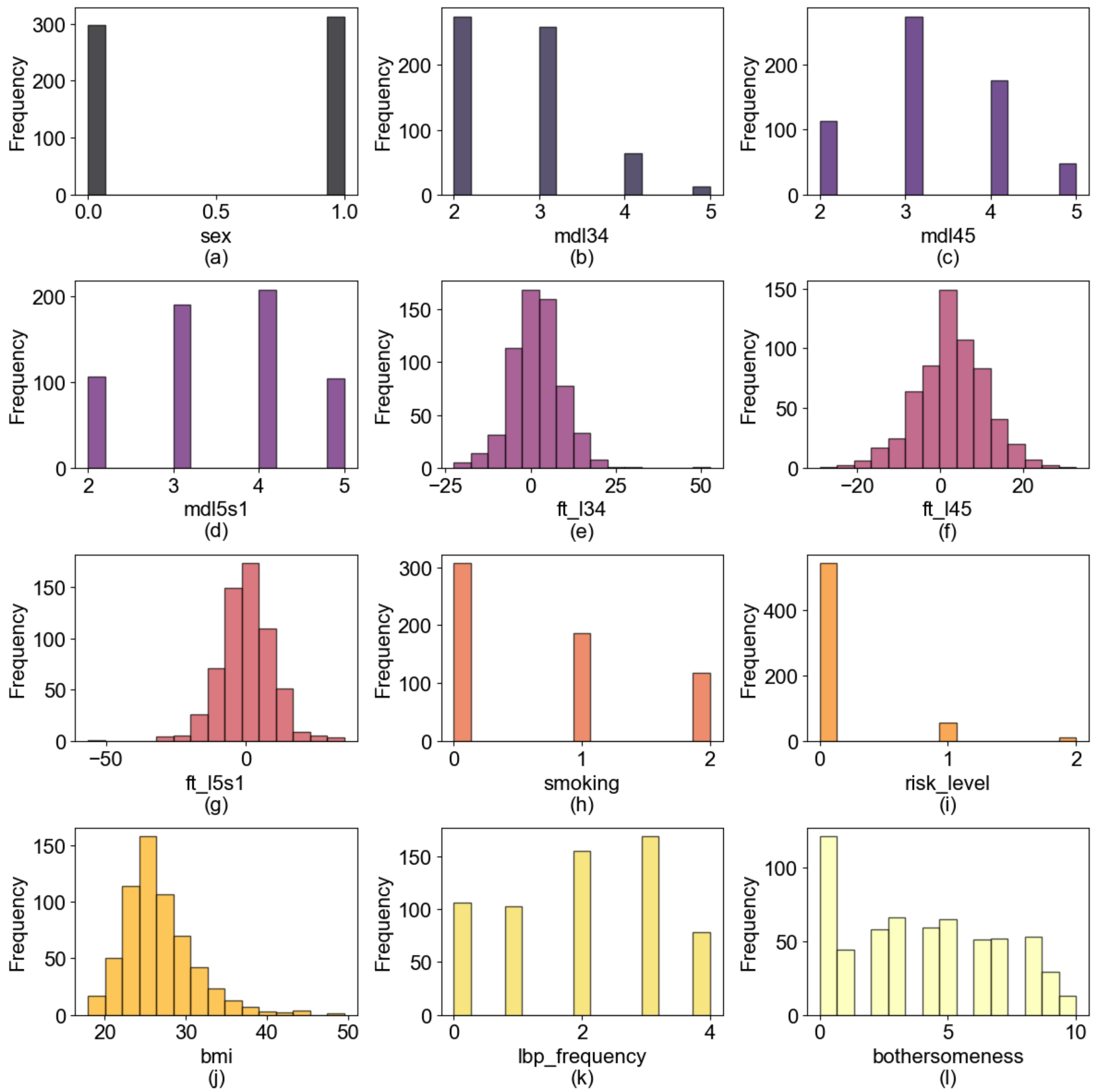

Supplementary Figure1: This figure presents the frequency distribution for all included dataset features: demographics: sex (a), bmi (j); disc degeneration (b, c, d) and facet tropism at vertebral levels L3/4 (e), L4/5 (f), and L5/S1 (g), lifestyle (smoking status) (h), self-reported low back pain frequency (k), pain-related bothersomeness (l), and the psychosocial risk score derived from the STarT Back Tool (i).

BMI: Body Mass Index;

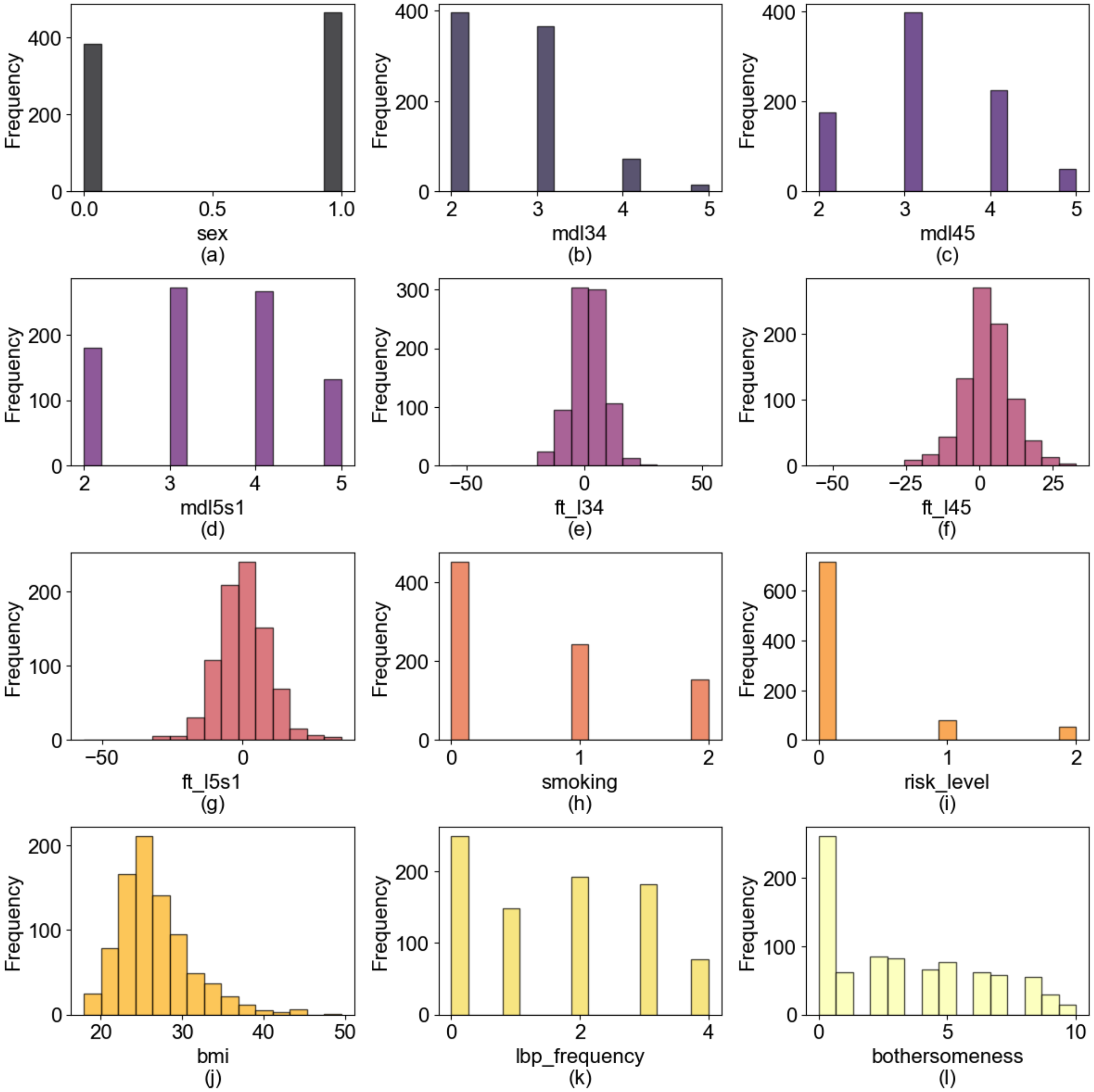

Supplementary Figure2: This figure presents the frequency distribution for all included dataset features: demographics: sex (a), bmi (j); disc degeneration (b, c, d) and facet tropism at vertebral levels L3/4 (e), L4/5 (f), and L5/S1 (g), lifestyle (smoking status) (h), self-reported low back pain frequency (k), pain-related bothersomeness (l), and the psychosocial risk score derived from the short version of the ÖMPSQ-Short(i).

ÖMPSQ-Short: Örebro Musculoskeletal Pain Screening Questionnaire - Short.

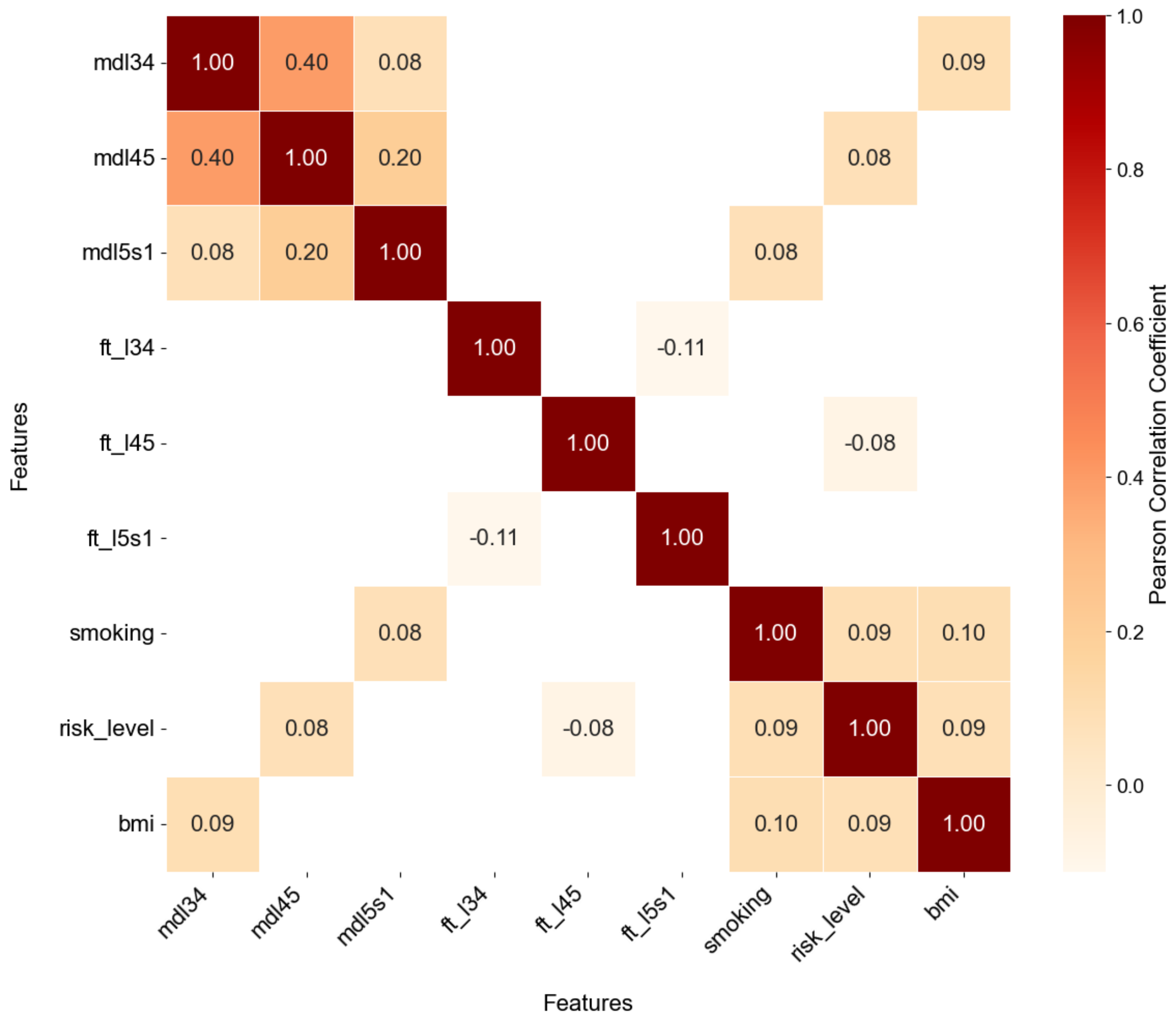

Supplementary Figure 3: Pearson Correlation Heatmap of Statistically Significant Variables. This heatmap displays Pearson correlation coefficients for variables from the SBT dataset that achieved statistical significance ($p < 0.05$).

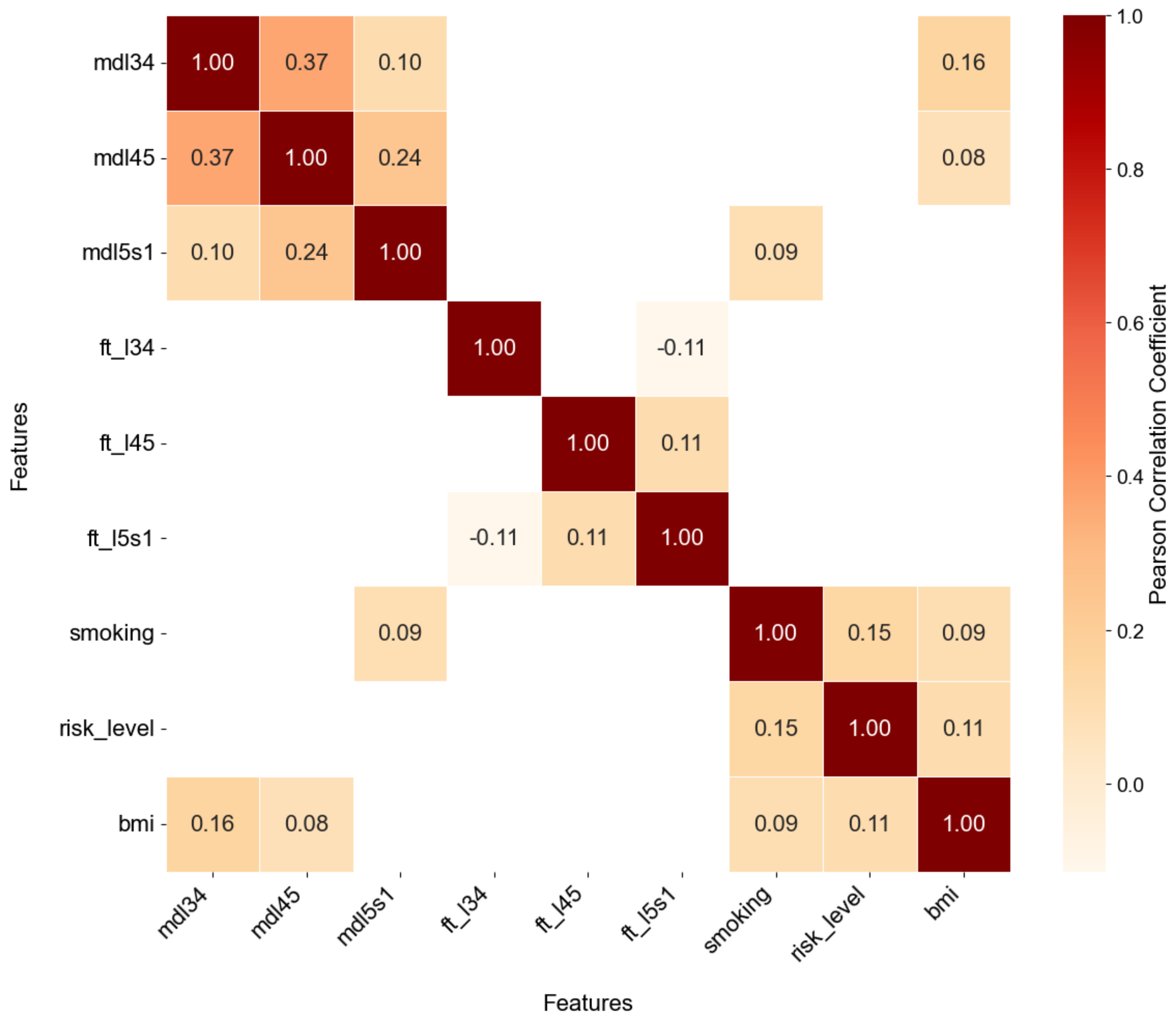

Supplementary Figure 4: Pearson Correlation Heatmap of Statistically Significant Variables. This heatmap displays Pearson correlation coefficients for variables from the ÖMPSQ Short dataset that achieved statistical significance ($p < 0.05$).

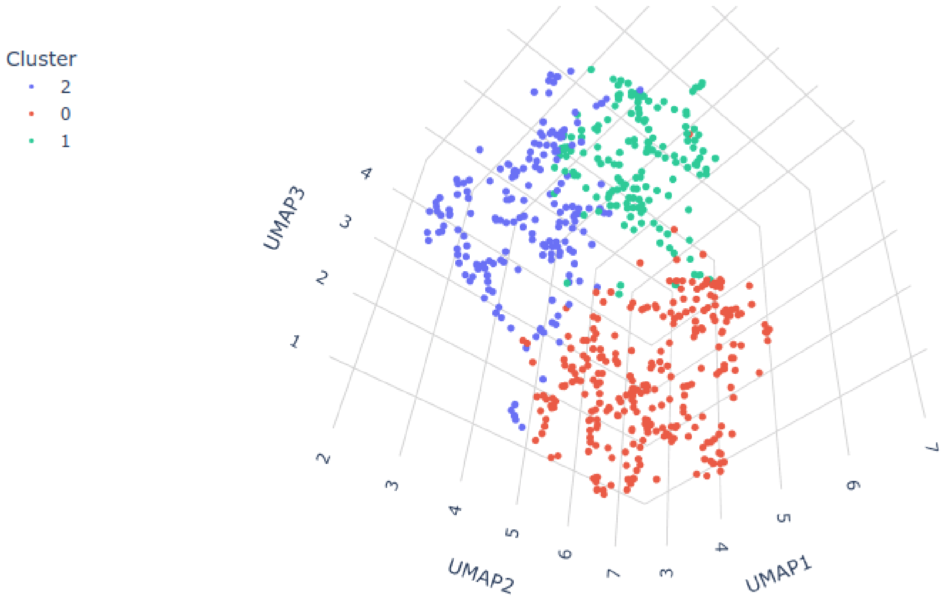

Supplementary Figure 5: 3D UMAP Visualization of SBT Data Clusters. This figure displays the K-means clusters identified within the SBT dataset, projected into a 3-dimensional space using UMAP. Distinct colors represent different cluster assignments.

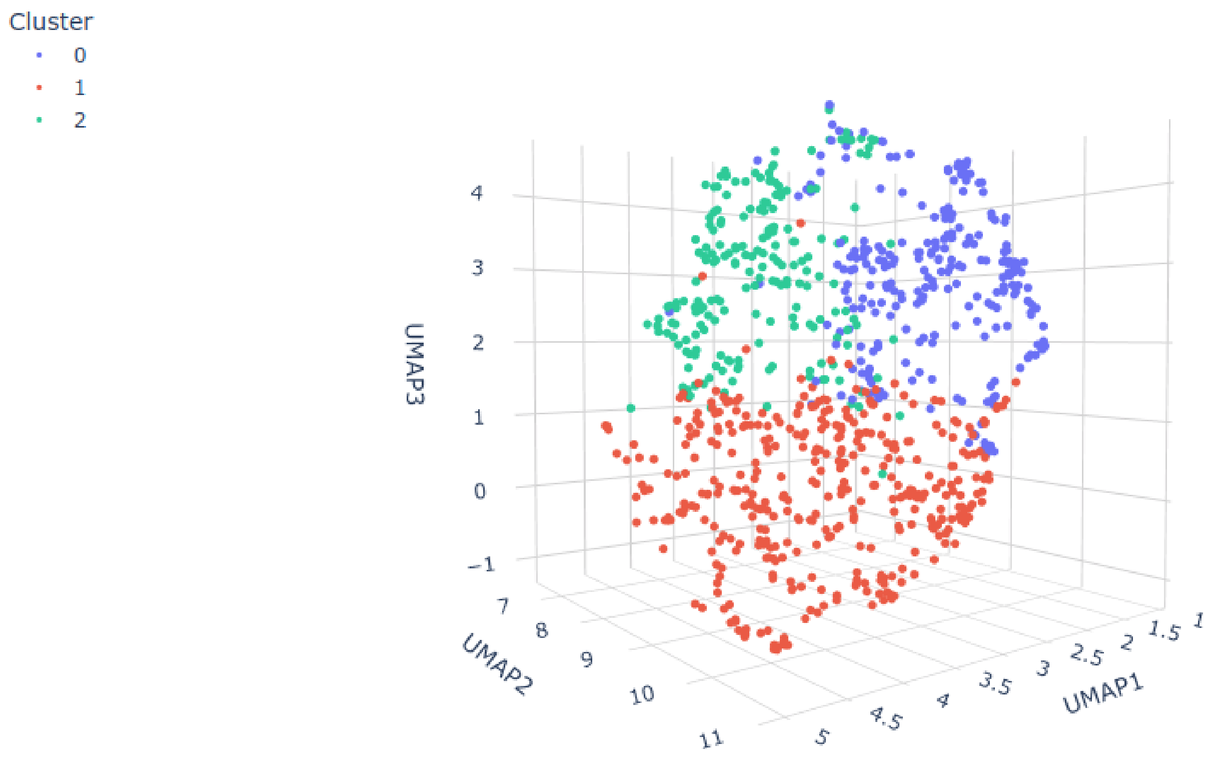

Supplementary Figure 6: 3D UMAP Visualization of SBT Data Clusters. This figure displays the K-means clusters identified within the SBT dataset, projected into a 3-dimensional space using UMAP. Distinct colors represent different cluster assignments.

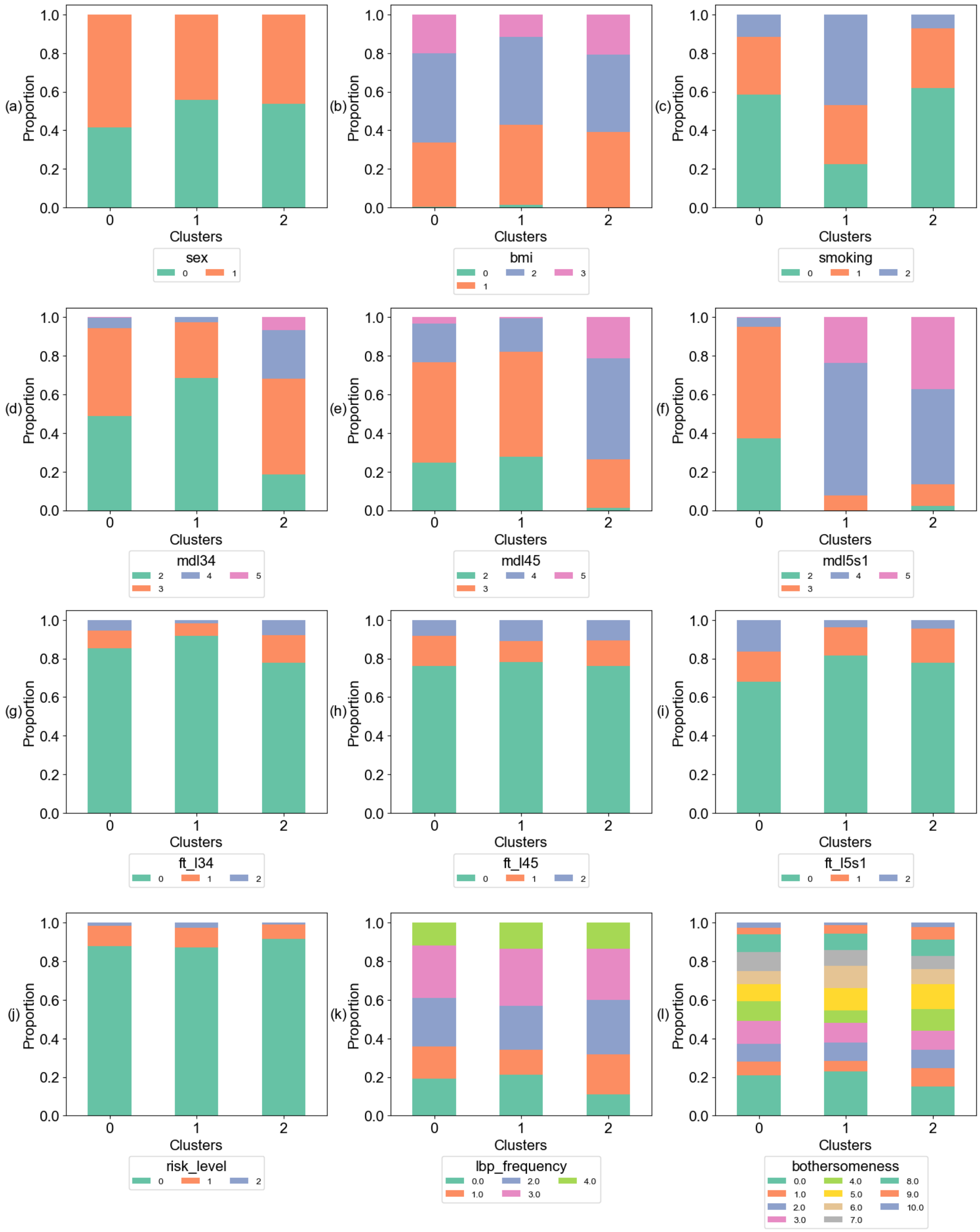

Supplementary Figure 7: Within-Cluster Feature Proportions for the SBT Dataset. This stacked bar plot illustrates the distribution of characteristics (a) sex 0:Male; 1:Female (b) bmi 0:Underweight; 1:Healthy Weight; 2:Overweight; 3:Obese (c) smoking status 0: Non-Smokers; 1:

Former Smokers 2: Current Smokers (d-f) Disc degeneration at L3/4, L4/5 and L5/S1 (g-i) Facet Tropism at L3/4, L4/5 and L5/S1 0: Normal; 1: Moderately Accentuated; 2: Severely Accentuated. (j) Psychosocial Risk computed from SBT 0: low risk ; 1: medium risk ; 2: high risk (k) self-reported low back pain frequency 0: No Pain; 1: 1-7 Days; 2: 8 to 30 days ; 3 : Greater than 30 Days but not daily; 4 : Daily (l) pain related bothersomeness scale of 1 to 10 where 10 is high for each cluster identified within the SBT data. Each bar represents a cluster, with segments indicating the proportion of different feature values within that cluster.

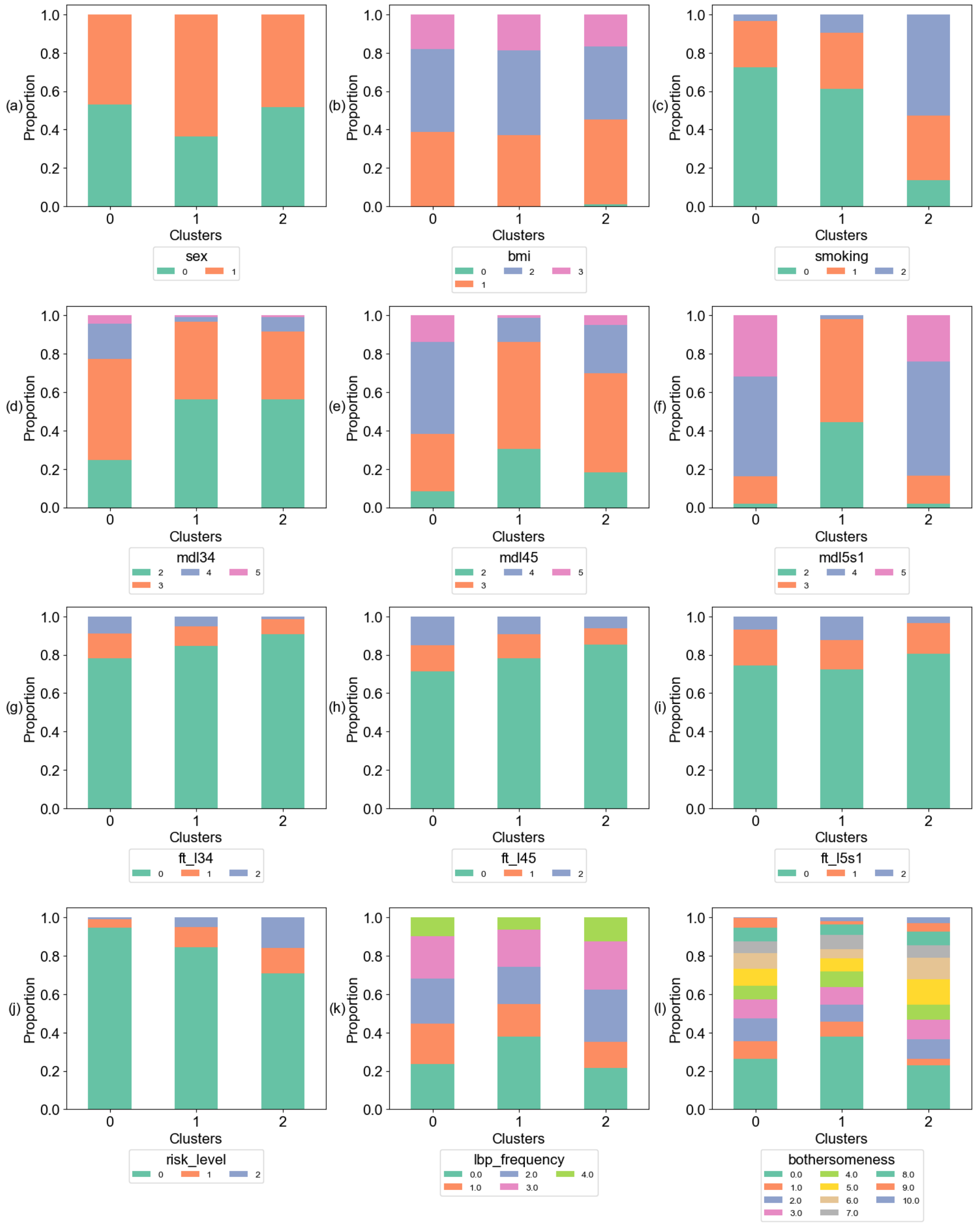

Supplementary Figure 8: Within-Cluster Feature Proportions for the ÖMPSQ Short Dataset. This stacked bar plot illustrates the distribution of characteristics (a) sex 0:Male; 1:Female (b) bmi 0:Underweight; 1:Healthy Weight; 2:Overweight; 3:Obese (c) smoking status 0: Non-Smokers; 1:

Former Smokers 2: Current Smokers (d-f) Disc degeneration at L3/4, L4/5 and L5/S1 (g-i) Facet Tropism at L3/4, L4/5 and L5/S1 0: Normal; 1: Moderately Accentuated; 2: Severely Accentuated. (j) Psychosocial Risk computed from ÖMPSQ short 0: low risk ; 1: medium risk ; 2: high risk (k) self-reported low back pain frequency 0: No Pain; 1: 1-7 Days; 2: 8 to 30 days ; 3 : Greater than 30 Days but not daily; 4 : Daily (l) pain related bothersomeness scale of 1 to 10 where 10 is high for each cluster identified within the ÖMPSQ Short data. Each bar represents a cluster, with segments indicating the proportion of different feature values within that cluster.

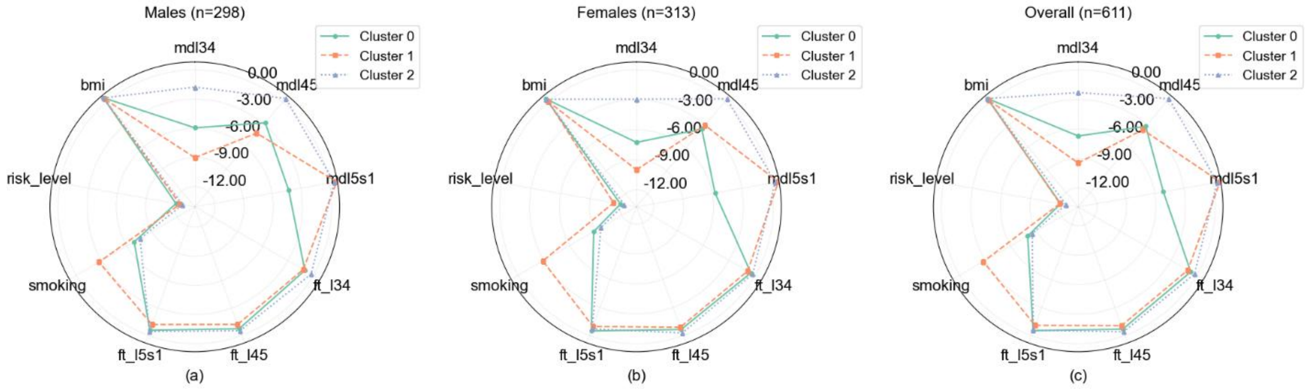

Supplementary Figure 9: Radar Plots Illustrating Feature Distributions Across Clusters for the SBT Instrument. These radar plots depict the mean feature values for each identified cluster derived from the SBT dataset. (a) radar plot characterizing clusters for sex=male (b) radar plot characterizing clusters for sex = Female and (c) radar plot characterizing clusters for both sexes. Each spoke represents a specific feature, and the polygons illustrate the average profile of each cluster.

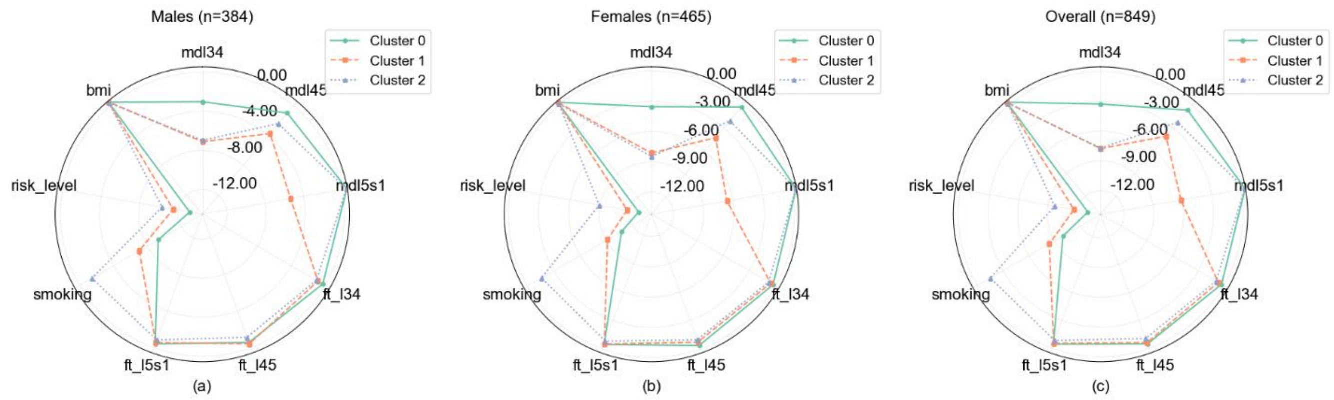

Supplementary Figure 10: Radar Plots Illustrating Feature Distributions Across Clusters for the ÖMPSQ Short Instrument. These radar plots depict the mean feature values for each identified cluster derived from the ÖMPSQ Short dataset. (a) radar plot characterizing clusters for sex=male (b) radar plot characterizing clusters for sex = Female and (c) radar plot characterizing clusters for both sexes. Each spoke represents a specific feature, and the polygons illustrate the average profile of each cluster.
